# The use of translation theory through reconciling between Englishisation and translanguaging by Arab instructors in EMI higher education classes: Training postgraduate students to be translators and interpreters

**DOI:** 10.3389/fpsyg.2022.1010704

**Published:** 2023-01-06

**Authors:** Rafat Y. Alwazna

**Affiliations:** Professor of Translation Studies, TESOL and Legal Translation, Department of European Languages and Literature, Faculty of Arts and Humanities, King Abdulaziz University, Jeddah, Saudi Arabia

**Keywords:** translation theory, Englishisation, translanguaging, EMI higher education, Arab instructors, postgraduate students, translation/interpreting training

## Abstract

Translation theory is taught at a postgraduate level in Arab countries through the use of English as a medium of instruction (EMI). It is often studied as a theoretical course, which is a major part of any EMI postgraduate translation/interpreting program taught in Arab universities. The present paper examines whether or not Arab instructors use translation theory when they train students in practical courses to be translators/interpreters in EMI higher education in a selection of Arabic-speaking countries in the Middle East. It also investigates whether or not translation theory, if used by Arab instructors in the translation/interpreting training at a postgraduate level, is utilized through English only or through translanguaging. It adopts a mixed method, consisting of a questionnaire, which has been distributed to 60 Arab translation/interpreting instructors/trainers from the selected Arab countries alongside three semi-structured interviews with three Arab professors who have been chosen from the 60 instructors for their long experience of translation/interpreting training. The paper argues that most of the participants use translation theory when training postgraduate students in practical courses to be translators/interpreters. This is because the use of theory offers students a solid ground for their problem-solving, decision-making and the ability to explain the logic behind their choice. The paper also shows that the participants do not completely adhere to English as a medium of instruction, rather they exercise translanguaging while using translation theory in practical courses. This is due to the specific translation/interpreting terminology contained in such theories, which may not be easily comprehended by students through the use of English on its own. Finally, the paper claims that the majority of the participants use multiple theories through translanguaging in their training. They argue that each theory has its own use and students should be exposed to as many theories as possible in EMI higher education in the selected Arab countries through translanguaging. This paper offers a baseline for using translation theory for the purpose of the translation/interpreting training through translanguaging in EMI higher education in the selected Arab countries, which may have implications for bilingual instruction of translation theory in other similar contexts.

## 1. Introduction

The field of translation studies has grown out of the need for translation teaching as well as translator and interpreter training. Indeed, translator and interpreter training is considered an important field categorized under the third branch of translation studies, which is known as ‘applied translation studies’ (Holmes, 1972/1988). Even though the topic of whether or not translation theory is used in the translator and interpreter training has long been tackled by a number of translation scholars, such as Levy (1965), Moser (1978, 1996), Komissarov (1985), Nord (1992), Kussmaul (1995), Mason (1998), Kiraly (2000), Shuttleworth (2001), Kelly (2005) and Lederer (2007), the present paper is different from all such studies as it particularly addresses the notion of whether or not translation theory is employed by Arab instructors in training students to be translators/interpreters in EMI higher education in a selection of Arabic-speaking countries in the Middle East. It also investigates as to whether or not translation theory, if used at all in practical courses, is exploited by Arab instructors for translation/interpreting purposes through Englishisation only or through translanguaging. Englishisation in the current context points to the use of English as the medium of instruction in employing translation theory for the purpose of the translation/interpreting training in practical translation courses and in all the materials specified for these particular courses in EMI higher education in the selected Arab countries. Translanguaging, on the other hand, refers in the current context to the use of Arabic together with English in employing translation theory for the purpose of the translation/interpreting training in practical translation courses and in all the materials specified for these particular courses in EMI higher education in the selected Arab countries. The Arabic-speaking countries in the Middle East on which the current research has been conducted are: Kingdom of Saudi Arabia, United Arab Emirates, Qatar, Oman, Bahrain, Iraq, Yemen, Jordan, Lebanon, Palestine, Egypt and Sudan. The reasons for conducting the current research on these specific countries stem from the fact that they are both Arab countries and use English in their postgraduate translation/interpreting programs or other related postgraduate programs that contain practical translation/interpreting courses.

The paper starts by showing the need for translation theory in training students to be translators/interpreters, presenting two opposing views, supporting the views of its proponents and refuting those of its opponents. A complete section is devoted to addressing EMI higher education in the selected Arab countries and whether Arab instructors use translation theory in training students to be translators/interpreters through the use of English only or through translanguaging. The paper then offers a succinct account of five different translation theories that are usually applied to translation/interpreting teaching and training. These are: literal translation, contrastive analysis, text-linguistics, information processing models and the interpretive theory of translation, respectively. The reason of addressing such theories lies chiefly in the fact that an important part of the paper in question is the translation/interpreting teaching/training. Moreover, Some of these theories can also be used as linguistic translation theories, as will be seen in section (6) where the participants have utilized a mixture of these theories and those for translation.

The paper seeks to answer four main research questions. These are: (1) Do Arab instructors make use of translation theory in training students to be translators/interpreters in EMI higher education classes in the selected Arab countries, and if so, why? (2) If Arab instructors make use of translation theory in training students to be translators/interpreters in EMI higher education classes in the selected Arab countries, do they adhere to Englishisation only, or do they reconcile between Englishisation and translanguaging, and why? (3) If Arab instructors make use of translation theory in training students to be translators/interpreters in EMI higher education classes in the selected Arab countries, how often do they use it? (4) What are the types of translation theory used by Arab instructors in training students to be translators/interpreters in EMI higher education classes in the selected Arab countries, and why?

To answer these research questions, a mixed method has been used. This typifies a comprehensive questionnaire which comprises both closed-ended and open-ended questions to elicit both quantitative as well as qualitative data and three semi-structured interviews to further obtain qualitative data. Sixty Arab instructors have been carefully chosen from different universities of 12 Arab countries in the Middle East to participate in the research questionnaire concerned. These participants have been asked to give their opinions concerning as to whether or not Arab instructors use translation theory in training students to be translators/interpreters in EMI higher education classes in the selected Arab countries, and if yes, how often they use it and the type(s) of theory they utilize with justification. In other words, the questionnaire has been distributed to elicit responses to answer the first, third and fourth research questions. Moreover, three eminent Arab professional translators/interpreters and translation/interpreting postgraduate instructors have been meticulously selected from the previous group of the 60 participants to be interviewed to give their own views on whether or not translation theory is used by Arab instructors in training students to be translators/interpreters in EMI higher education classes in the selected Arab countries and whether or not translation theory is utilized by Arab instructors in training students to be translators/interpreters in EMI higher education classes in the same countries through Englishisation only or through translanguaging, supporting their views with justifiable reasons. In other words, the interviews have been conducted to answer the first and second research questions. The present paper argues that all the interviewees and the majority of the Arab instructors who have participated in the current questionnaire make use of translation theory in training students to be translators/interpreters in EMI higher education classes in the selected Arab countries. This is because the use of translation theory helps trainee students accomplish their practical translation tasks properly. It offers them a solid ground for their problem-solving, decision-making and the ability to explain the logic behind their choice. The paper also shows that although all the interviewees exploit translation theory when training students to be translators/interpreters in EMI higher education classes in the selected Arab countries, they do not wholly stick to English as a medium of instruction, rather they exercise translanguaging. This is owing to the fact that certain translation theories are difficult to understand by trainee students through Englishisation only. This is chiefly due to the specific translation/interpreting terminology contained in such translation theories, which may not be easily comprehended by students through the use of English as the only medium of instruction. Finally, the present paper claims that the majority of the Arab instructors who have participated in the current questionnaire always use translation theory and indeed exploit multiple translation theories in their training, among these are: literal translation, contrastive analysis, text-linguistics, information processing models, the interpretive theory of translation, communicative translation, skopos theory, polysystem theory, functional theory, cultural turn and sociolinguistic theory. The participants justify their use of multifarious theories by stating that each theory has its own use and trainee students should be exposed to as many theories as possible. Furthermore, the use of any theory is contingent upon different factors, such as text type, type of audience and cultural considerations.

## 2. The need for translation theory in training students to be translators/interpreters

A number of professional translators claim vehemently that translation/interpreting training does not require translation theory, nor is translation/interpreting training indeed necessary (Baker, 1992, p. 3; Robinson, 1997, p. 175–176). This applies to all types of training whether received in professional or higher education institutions. Nonetheless, they are of the opinion that the optimum way for preparing students to be translators and interpreters is to enable them to join the profession where they spend a period working within the professional environment. Furthermore, they need to be aware of the required technical knowledge and be abreast of the necessary aptitudes (Shuttleworth, 2001, p. 498). The extensive practice of translation/interpreting by trainee students does not qualify them to be professional expert translators/interpreters. Indeed, translation is considered an intricate operation, and theory plays a crucial role in regulating and generalizing translation problems (Lederer, 2007, p. 33). This may also apply to interpreting, albeit to a lesser extent. This may probably be due to the fact that interpreting, compared to translation, requires less theory and more practice. Ingo (1992, p. 49) asserts that translation theory can fulfill the same function concerning translation and interpreting as that fulfilled by grammar concerning languages. Within the training process, trainee students are allowed to enjoy some freedom, not adhering to the specific detail of the source text and playing their roles with regard to the text, its components, its reality and so on. Their particular approach to translation problems will ipso facto enhance the sense of self-assurance among themselves. Hence theory can be utilized by instructors/trainers to direct trainee students to the way of deep and proper thinking and offer them certain principles that can scaffold them in the process of decision-making (Lederer, 2007, p. 33). Along similar lines, Komissarov (1985, p. 208) contends that it is not of the tasks performed by translation theory to provide the translator with well-thought out solutions to overcome his/her problems. Theory can never replace deep thinking or decision-making. This undoubtedly applies to the context of instructors employing translation theories in their teaching.

The need for theoretical framework in training students to be translators/interpreters in EMI higher education can be viewed from two different perspectives: enhancing trainee students’ declarative knowledge (knowing what) and strengthening their procedural knowledge (knowing how) (Wilss, 1998). Instructors/Trainers are required to make use of the results of translation/interpreting research in two ways. They need to exploit such results in a direct way; they should be fully aware of the theoretical details that need to be taught to trainee students, so that they can train their students to be highly efficient translators and interpreters who can perform formidable translation/interpreting tasks as well as act professionally as translators and/or interpreters. Instructors/Trainers should also utilize the findings of translation/interpreting research indirectly, i.e., they should be conversant with the optimum way in which their teaching should be delivered (Kiraly, 1995, 2000), as will be discussed in section (4) below.

It can be argued that translation/interpreting practice itself involves particular difficulties, so does translation/interpreting training. The difficulties particular to the former stem broadly from the linguistic and cultural differences between the source and target languages, while the difficulties specific to the latter reside in training students on how to translate/interpret particular texts, taking into account such differences. Also, translation/interpreting training is concerned with how students are trained to adopt appropriate translation/interpreting techniques/strategies for their tasks on the basis of the text type, purpose of the translation/interpreting and the type of audience. Hence comes the relevance of translation theories to translation/interpreting practice, and therefore to translation/interpreting training. Translation theories help facilitate the job of translation/interpreting practice and training in regulating and generalizing translation problems, as claimed by Lederer (2007). They set out rules for the translators/interpreters to identify and regulate translation obstacles. This would, with no doubt, help translation/interpreting practitioners adopt the optimum technique(s) to minimize translation loss.

## 3. EMI higher education: Englishisation versus translanguaging

English is considered the most widely-spread and first global language (Crystal, 2003, p. 1; Alwazna, 2020, p. 571). Within the academic sphere, English was known to have been among the few languages used extensively in academia. Indeed, Tim Johns is believed to have coined the term ‘English for Academic Purposes’ (EAP) in 1974, which was first used in a published collection of papers edited by Cowie and Heaton in 1977 (Jordan, 2002; Alwazna, 2010, p. 9). When the journal of *English for Specific Purposes* (ESP) was established in 1980, EAP was regarded as one of its two branches, while the second branch was typified by the use of language in professional and workplace settings, known as ‘English for Occupational Purposes’ (EOP) (Hyland, 2006, p. 2; Alwazna, 2010, p. 9). EAP is defined as: “teaching English with the aim of assisting learners’ study or research in that language” (Hyland, 2006, p. 1). Since then, teachers and students, whether native or non-native speakers of English, had started to use EAP in their writing in their different and diverse academic disciplines. Indeed, EAP is viewed as a broad term, which comprises undergraduate and postgraduate teaching, administrative practices, classroom interactions, student writing and research genres (Hyland, 2006, p. 1; Alwazna, 2010, p. 9).

In the English-speaking countries, course designers and providers have recognized the difference in needs between teaching students English to be used for their academic studies and teaching those who aim to use English for different purposes. In these countries, most of the foreign students whose native language is not English, including those from the countries under study, start learning EAP through joining pre-sessional courses, which are chiefly designed to improve students’ communicative skills and academic competence to reach the level of English proficiency required for entry into EMI universities (Hyland, 2006, p. 4; Alwazna, 2010, p. 9).

The countries under study are categorized within the Expanding Circle in which English is regarded as a foreign language, but gains high importance and recognition in multifarious respects, as classified by Kachru (1985). In the selected Arab countries, there has been an exponential growth of EMI postgraduate programs in several higher education institutions in the last two decades. This may emanate from the fact that higher education policy makers in these countries seek to internationalize their higher education programs for the sake of having academic and research collaboration with the Western universities, raising university ranking and increasing the number of international students. This rapid growth of EMI higher education programs in the selected Arab countries runs in line with the evidently increased use of English in Saudi Arabia and the rest of the countries under study due to the current policies followed in these countries, which were predominantly influenced by globalization and modernisation (Mahboob, 2013; Mahboob and Elyas, 2014; Alwazna, 2020, p. 572). Moreover, as Elyas et al. (2020) argue, embracing English is highly necessary in Saudi Arabia, as it is in all the countries under study, for having a strong economy and achieving the Saudi Vision 2030. What is more, due to the presence of expert foreign teaching staff in different higher education institutions in the countries under study, including Saudi Arabia, English has become used as the medium of instruction in such institutions (Elyas et al., 2020). However, as language and culture are inextricably linked to one another and are never inseparable (Faiq, 2004; Alwazna, 2014a, 2017), English used in the Middle East is characterized by local practices and specific patterns of use that are different from formal English (Mahboob, 2013). Alrawi (2012) attributes the variation between English used in Saudi Arabia and formal English to the interference of Arabic. The same conclusion has been reached by Fallatah (2017), who reports that the comedians in her research study have made use of non-standard English intentionally to imitate an aged woman with a low level of English proficiency.

In the countries under study, MA programs in translation/interpreting are often taught in English. This applies to theoretical courses included therein, such as translation theory, translation/interpreting strategies/techniques and so on. It also applies to practical courses, such as applied translation/interpreting, translation technology and so on. Such monolingual teaching technique has long gained acceptability within the context of the direct method and has continued to exercise influence on multiple language teaching approaches for more than a century (Yu, 2000). Unfortunately, to my knowledge, TESOL has not adopted an upright stance as to whether or not English teaching or teaching other academic content in EMI bilingual higher education classes should be carried out entirely with the use of English. However, such issue surfaced in the *TESOL Quarterly* and other outlets in the 1990s (Phillipson, 1992; Auerbach, 1993; Lucas and Katz, 1994). It has increased momentum in recent years (Manyak, 2004; Cummins, 2007; García, 2008). However, switching from English into Arabic, or what is termed as translanguaging is exercised by Arab instructors when explaining specific theories, approaches and methods specific to translation/interpreting praxis, as will be shown in section (6) below. This is lent credence by Cook (2001), who argues over the merit of judicious use of the native language in teaching second/foreign languages, but warns, at the same time, that in spite of the permissibility of employing the native language under certain circumstances, the second/foreign language should be extensively used (p. 413). This is advocated by García (2008), who argues for the use of translanguaging or bilingual instructional strategies in classrooms. Along similar lines, it is known that one important fundamental principle of learning rests chiefly upon the fact that the preexisting knowledge of the learner represents the basis of all of his/her future learning (Bransford et al., 2000). With this in mind, since the preexisting knowledge of English learners is encoded in their native language, building on such prior knowledge demands linking new concepts in English to the learners’ first language cognitive schemata (Lucas and Katz, 1994; Cummins, 2001, 2007, 2009; García, 2008). Such linking requires the use of learners’ mother tongue in the classroom to be done effectively (Cummins, 2009). Switching from English into Arabic in such EMI higher education translation/interpreting programs in the selected Arab countries may be due to different factors. One of these factors is that certain theoretical and practical courses peculiar to MA translation/interpreting programs may contain certain terminology that cannot be fully comprehended by students with the use of English as the only medium of instruction. Hence Arab instructors resort to Arabic to ensure full comprehension of such terminology by students. Malakoff and Hakuta (1991, p. 163) point out that translation offers a flexible method of raising linguistic awareness and pride in bilingualism. Arab instructors may also switch into Arabic to show students the practical part of any theory, method, approach and so on they explain. This is to show students how such theoretical framework is applied to translation/interpreting practice.

## 4. Translation theories applied to translation teaching

In the middle of the 20th century, when the field of translation studies began to receive more attention by scholars and students, several translation theories have come into light, and such theories are presently being made use of in translation teaching (Lederer, 2007, p. 16). The foundations on which translation students are trained vary depending on the type of theory adopted by instructors/trainers. It is argued that a teaching theory should offer a coherent view with regard to the way in which translation is accomplished, not a prescriptive account of true-false approach or detail which is not based on facts. The translation/interpreting instructor who is well versed in the mental processes required for translation and interpreting will barely employ prescriptive translation teaching theory (Moser, 1996, p. 201). Teaching translation is inevitably grounded in theoretical assumptions that are concerned with the nature and the way in which translation is accomplished. Despite the fact that not all the teachers of translation are cognisant of their own theoretical assumptions, certain theories are lucidly adopted within the field of translation training (Lederer, 2007, p. 17–18). Although there are opposing views concerning whether or not a course on translation theory is necessary in the curriculum in the translation/interpreting teaching/training, it is claimed that a single theory should be selected as a basis on which translation teaching is carried out in a given context (Lederer, 2007, p. 18). Indeed, the majority of postgraduate translation/interpreting programs, including those taught in the countries under study, comprise translation theory as a course in their study plan. Consequently, expert instructors/trainers who train novice translators/students should receive sufficient training in the use of translation theory and related fields to be qualified for teaching translation. For instance, Kussmaul (1995, p. 2-3) introduces a book entitled: *Training the Translator*, where he aims at exploring multiple aspects of translation methodology, drawing on psycholinguistics, speech act theory, text-linguistics, text typology as well as functional sentence perspective. He hopes that the content of his publication is beneficial to translation teachers when teaching. On the contrary, he does not recommend that the translation teachers be conversant with the numerous fields he has addressed. Based on the foregoing, there are undeniably patent differences between the translation teacher and the translation scholar whose job is to contribute to the pool of translation studies (Lederer, 2007, p. 19). In crude terms, instructors/trainers should be conversant with the optimum way in which their teaching should be delivered. This includes applying the most appropriate teaching methods, such as enabling their students to translate in groups, imposing project work and so on (Kiraly, 1995, 2000), choosing teaching materials (Asensio, 2003), designing curriculum (Kelly, 2005), assessing the translation quality (Mossop, 2001) and developing translation tools, such as translation memories, online dictionaries, translation corpora and so on (Austermuhl, 2001).

Linguistic translation theories have been divided into three major approaches, all of which have been and are still used in translation pedagogy. One of such approaches is literal translation, which was deemed the linguistic theory of 1960s and early 1970s, which analyzed the sentence as its maximum unit and represented at that time the point of reference for the field of translation (Snell-Hornby, 1992, p. 21). The impact of literal translation on translation training/teaching has persisted and still exists as a result of what is known as ‘legacy of language teaching’ (Colina, 2002, p. 1). It is claimed that the historical foundation of grammar translation methods is closely linked to linguistic theories, such as structuralism, which is characterized by placing special emphasis on form, such as phonology and morphology, with little or no focus on the communicative functions of language (Colina, 2002, p. 2). It is the approach that translation students who join translation programs are seen to have been accustomed to the use thereof, though it has been heavily criticized for the negative effect it has on students’ attitudes in the classroom.

The second approach to translation teaching is known as contrastive analysis, which is still in use, particularly in translation teaching at the university level, including EMI postgraduate classes (Lederer, 2007, p. 20). Advocates of this approach are likely to hinge upon Vinay’s and Darbelnet’s (1957, 1998) work in comparing French to English. The approach of contrastive analysis is considered limited to the language level and is ipso facto employed in the context of language teaching, though it does not cater for translation creativity. Thus, it may not be ideally applicable for translation teaching (Lederer, 2007, p. 20). The third and most recent approach of the three is known as text-linguistics, which carefully examines the use of language in communicative situations. It is deemed the optimum approach insofar as it draws a comparison between the original and target texts in view of seven standards of textuality (Beaugrande and Dressler, 1981), these are: coherence, cohesion, informativity, acceptability, intentionality, situationality as well as intertextuality (Lederer, 2007, p. 20). It is claimed that text-linguistic approach tends to link textual features to the socio-textual practices of the speech communities concerned and to the motivations as well as purposes specific to both text producers and recipients (Mason, 1998, p. 64).

Hatim and Mason (1997) accentuate the importance of rejecting the approaches that are characterized by haphazard curriculum design for translation students training. Their approach to material selection for translation students training is evidently grounded in text-linguistics. Nonetheless, if text-linguistics is applied in an incorrect way to translation training, it may give rise to the fact that translation is reclaimed by linguistics, which would require linguistic training prior to translation training as well as acquaintance with terminology, which is both heterogeneous and complicated (Salama-Carr, 1990, p. 168). The fledgling translator and translation student may make mistakes with regard to lexis and misunderstand the meaning of the text, reconsidering the word the main translation unit. With this in mind, the focus given to the comparison between the original and receptor texts may look more relevant for student assessment and translation criticism than for translation training (Lederer, 2007, p. 20). The main principle governing any process of translation lies chiefly in the purpose of the complete translation action, according to the functionalist theory (Nord, 1997, p. 27). Considering such theory for translation teaching, this may run the risk of making trainee students feel that they have been given a leeway in the translation of a text as such theory prevents the influence of the source text on the translation. This is the reason behind the addition of the concept of ‘loyalty’ made by Nord (1992, p. 41), which restores adherence to the original text, although this may not look necessary as long as its surface features are concerned. The advantage of this theory resides mainly in the fact that text analysis is not limited to intratextual factors, rather it extends to cover extratextual factors the analysis of which precedes the process of reading the text as the situation always comes before textual communication (Nord, 1992, p. 43). The translator draws a comparison between the results of analyzing the source text and those of analyzing the translation purpose. Based on such comparison, the translator will be in a good position to decide how and in what extent the original text needs to be adapted to fit the target language communicative situation and what strategies should be adopted to arrive at an adequate and coherent target text (Nord, 1997, p. 45).

Another theoretical framework used, particularly in simultaneous interpreting lies essentially in information processing models, which have been developed, comprising ‘complex multi-stage serial accounts’ (Setton, 1999, p. 34). Certain aspects of this theory have been applied to interpreting students training (Moser, 1978). Another theory that has been developed, with propensity toward translation students training is represented by the interpretive theory of translation, which is viewed by Nord (1997, p. 39) as being useful for functionalist approaches. The interpretive theory of translation caters for the function of both the original and target texts, takes account of the generic psychological processes specific to the comprehension and production of discourse and accentuates the task performed by translators in conveying sense across language boundaries. This, alongside the methodology for performing the task properly, are what this theory seeks to convey to translation trainee students (Lederer, 2007, p. 21). Having considered sense the main foundation of a particular theory of translation teaching might be regarded as simplistic or defective, however, training programs grounded in the interpretive theory are highly reputable and have proved fruitful (Brisset, 1993; Lavault, 1999; Setton, 1999). Trainers’/instructors’ use of psychological principles helps them guide students to the way in which sense can be comprehended, i.e., they help students become able to place themselves in a position to comprehend both the interpreted speech and the translated text. Moreover, trainers/instructors need to gain an understanding of certain rhetorical and linguistic rules, which mainly typify the differences between discourses and systems particular to different languages in order to provide their students with the methods required for the stage of reformulation (Lederer, 2007, p. 22). Finally, it is noteworthy that the ultimate goal for the translation and interpreting training is to show trainee students the way in which they can build their own theories and help them think in a more constructive way (Robinson, 1997, p. 182).

## 5. Methodology

The present paper makes use of a mixed method to collect both quantitative as well as qualitative data. This is represented by both a comprehensive questionnaire and three semi-structured interviews. The selection of the participants has been carefully made. About 60 Arab instructors/trainers have been meticulously chosen to participate in the questionnaire concerned. Three full professors of the 60 Arab instructors/trainers who have been working as translation/interpreting instructors/trainers for more than 10 years, have been interviewed. The sampling method used in the paper concerned is a random sampling method, known as a stratified random sampling method. Such sampling method is deemed representative as it is based on specific strata, which serve as pre-determined characteristics; namely: (1) All the participants are Arab university instructors/trainers of translation/interpreting. (2) They all teach in universities located in the selected Arab countries. (3) They all teach practical/training courses of translation/interpreting, which are part of EMI postgraduate translation/interpreting programs or other related EMI postgraduate programs that comprise practical translation/interpreting courses. The 60 participants who belong to 12 Arab countries, as stated in section (1), teach practical translation/interpreting courses at a postgraduate level in different universities in the countries under study. Six of them teach in Effat University in Kingdom of Saudi Arabia, while the same number of participants teach in the University of Sharjah in United Arab Emirates. Six teach in Hamad Bin Khalifa University in Qatar, six teach in Dhofar University in Oman, six teach in University of Bahrain in Bahrain, six teach in University of Baghdad in Iraq and six teach in University of Science and Technology in Yemen. Six participants teach in the University of Jordan in Jordan, six teach in American University of Beirut, six teach in An-Najah National University in Palestine, six teach in Ain Shams University in Egypt and six teach in the University of Khartoum in Sudan. The three full professors who have been chosen from the 60 Arab instructors/trainers for interviewing teach in Hamad Bin Khalifa University in Qatar, University of Baghdad in Iraq and the University of Jordan in Jordan, respectively. The representativeness of such stratified random sampling method also stems from the fact that it yields reliable and generalisable results specific to the strata governing the choice of the sampling of this method.

The questionnaire consists of eight questions; three of which are completely closed-ended questions, while five are partly closed-ended and partly open-ended questions, which has been distributed online through Google forms to elicit both quantitative as well as qualitative data. The first question enquires about the participant’s gender, whereas the second investigates as to whether the participant is a translation instructor/trainer, interpreting instructor/trainer or both. The last closed-ended question asks about the participant’s length of experience working as a translation/interpreting instructor/trainer for postgraduate students. The last five questions are partly closed-ended and partly open-ended where the first question seeks to take the participant’s views with regard to whether or not translation/interpreting from and/or into Arabic demands the use of translation theory with justification. The second asks as to whether or not the participant uses translation theory in the translation/interpreting training with justification. The last three questions should only be answered by Arab instructors/trainers who employ translation theory in their training. In other words, these questions are related to the use of translation theory in the translation/interpreting training and should ipso facto only be answered by those who use it in their training. The first enquires about the participant’s frequency of using translation theory in the translation/interpreting training with justification, while the second investigates as to whether or not the participant’s use of theory is contingent upon text type with justification. The last question asks the participant to specify the translation theory(s) he/she utilizes in the translation/interpreting training with justification.

On the other hand, the interviews are composed of two questions, which are both closed-ended and open-ended to further elicit quantitative and qualitative data. The first question seeks to investigate as to whether or not the interviewee uses translation theory in the translation/interpreting training, with justification. This question may seem repetitive as it has already been raised in the questionnaire and the interviewees have been chosen from the questionnaire participants. However, in the interviews, the interviewee may have a better chance to express his/her views in a more detailed way than that of the questionnaire. This, with no doubt, will enrich the data and enhance the quality thereof. The second question of the interviews enquires about whether the interviewee adheres completely to Englishisation or practice translanguaging when using translation theory in the translation/interpreting training, with justification. Tables of numbers and percentages have been adopted to present the quantitative data in all the 10 questions of both the questionnaire and interviews concerned. The percentages used in the first eight tables indicate the number of the questionnaire participants out of 100, taking into account the specified 60 questionnaire participants. On the other hand, the percentages in the last two tables point to the number of the interviewees out of 100, taking into consideration the specified three interviewees. When analyzing the qualitative data of the last five questions of the questionnaire and those of the interviews, the participants will be grouped on the basis of the similarity and approximation in opinion concerning a particular concept, with the use of phrases like: ‘all, the majority, most of, one of, the minority, a group of participants, another group of participants, etc.,’

## 6. Data analysis

As a response to the first question, Table 1 shows that 53.3% of the participants who have taken part in the questionnaire in question are males, while 46.7% of the participants are females. Answering the second question, Table 2 demonstrates that 60% of the participants who have contributed to the questionnaire concerned consider themselves translation instructors/trainers, whereas 40% of the participants consider themselves both translation and interpreting instructors/trainers. No record of any participant who considers himself/herself an interpreting instructor/trainer in Table 2. Responding to the third question, Table 3 shows that 50% of the participants who have taken part in the questionnaire under study have been working as translation/interpreting instructors/trainers for postgraduate students for more than 10 years, while 30% of the participants have been working as translation/interpreting instructors/trainers for postgraduate students for more than 5 years. About 16.7% of the participants have been working as translation/interpreting instructors/trainers for postgraduate students for more than a year, whereas 3.3% of the participants have been working as translation/interpreting instructors/trainers for postgraduate students for more than 3 years. No record of any participant who has been working as translation/interpreting instructor/trainer for postgraduate students for less than a year in Table 3.

**Table 1:** The percentage of the participants’ gender.

Gender	Percentage
Male	53.3%
Female	46.7%

**Table 2:** The percentage of the participants’ teaching/training specialization.

Teaching/training specialization	Percentage
Translation instructor/trainer	60%
Interpreting instructor/trainer	0%
Both	40%

**Table 3:** The percentage of the participants’ length of experience.

Length of experience	Percentage
Less than a year	0%
More than a year	16.7%
More than 3 years	3.3%
More than 5 years	30%
More than 10 years	50%

As Table 4 shows, the participants who think that translation/interpreting from and/or into Arabic requires the use of translation theory are 83.3%, while those who do not think that translation/interpreting from and/or into Arabic requires the use of translation theory are 16.7%. Therefore, the majority of the participants believe that translation/interpreting from and/or into Arabic demands the use of translation theory. One group of them hold the view that translation theory should be adopted in translation/interpreting from and/or into Arabic as Arabic and English, for instance, employ different forms to convey meaning. They claim that the use of translation theory in translation/interpreting helps relay the optimum meaning. They go on to argue that translators/interpreters make use of translation theory in overcoming the problems they encounter during the translation/interpreting process. Hence it helps them accomplish their job properly. Another group of participants support the use of translation theory in translation/interpreting, arguing over the merit of the impossibility to achieve proper translation without taking into consideration translation theories. They assert that translation theory serves as a basis for decision-making and allows for methodical processes to be replicated. Within the same line of thought, a third group of participants, lending credence to the use of translation theory in translation/interpreting, state that translation theory may result in more informed translation decisions. They point out that theory informs practice where practice-based theory as well as practice should run in line with translation training and teaching. They hold the view that theory is regarded as a foundation on which translation decisions and techniques are primarily grounded. Hence translators/interpreters view translation theory as a good basis for their decision-making alongside their talent. This is advocated by the fourth group of participants who are of the opinion that using theories may scaffold the translators/interpreters in justifying their chosen decisions. They add that theories are deemed the output of practice. Therefore, translators/interpreters should be cognisant of certain appropriate theories to enhance their translation/interpreting tools. The last group of participants who are of the belief that the use of translation theory is required in translation/interpreting claim that translation is a theory-based process pertaining to the notion of abstracting the intended meaning of a particular text from its forms and reproducing this meaning in the receptor language. So, such process is composed of examining equivalence, transposing grammatical structure, building communicative situation and understanding cultural context of the source language text. They go on to affirm that translation theory is needed to comprehend the way in which the source text is decoded into the target text, particularly when cultural adaptation is demanded. Hence translation theory is viewed as a generic frame of a linguistic transfer between languages. Moreover, translation theory is used due to the cultural and ideological differences between languages.

**Table 4:** The participants’ attitudes toward the necessity of the use of translation theory in translation/interpreting from and/or into Arabic and their percentages.

Participants’ attitudes	Percentage
The participants who think that translation/interpreting from and/or into Arabic requires the use of translation theory	83.3%
The participants who do not think that translation/interpreting from and/or into Arabic requires the use of translation theory	16.7%

By contrast, the minority of the participants do not believe that translation/interpreting from and/or into Arabic requires the use of translation theory. One group of them claim that there is no need for translation theory for the purpose of translation/interpreting, though learning such theory is desirable by some translators/interpreters. Another group of participants believe that translation theories slightly influence translation practice and hardly optimize translation quality. The last group of participants argue that translation theory is pointless and that practice makes perfect. They claim that translation activity is all about language skills and has no direct relation to theories.

As shown in Table 5, the participants who use translation theory in the translation/interpreting training are 86.7%, while those who do not use translation theory in the translation/interpreting training are 13.3%. Hence most of the participants make use of translation theory in the translation/interpreting training in EMI higher education classes in the selected Arab countries. One group of them hold the view that they use translation theory in training insofar as it helps trainee students achieve their practical translation tasks properly. They further add that one pivotal reason behind their use of translation theory in training students springs from the fact that it enables trainee students to have a solid ground on which their translation decisions can intrinsically be based. Along similar lines, another group of participants contend that they adopt translation theory in their training as it is fruitful in the process of problem-solving. They go on to assert that translation theory serves as a useful guide for trainee students, facilitating the training process and simplifying its steps as theory is grounded in evidence and cumulative experiences over decades. A third group of participants, seconding the use of translation theory in training students, justify their use by claiming that the use of theory in training enables the trainee students to compare theories and established sciences to real-life practices. They add that theory helps trainee students explain the rationale and logic behind any decision they undertake. They continue to claim that translation theory paves the way for trainee students to measure and weigh possible solutions and then choose the most appropriate one. The fourth group of participants refer their use of translation theory in training students to the fact that translation/interpreting is deemed a science with specific principles and theories that students should be trained to apply. They explicate that the use of translation theory helps trainee students preserve the intended meaning in the target text with the use of the appropriate form for the target language. It also scaffolds them in identifying the differences between different alternatives. Indeed, instructors/trainers should use translation theory in their training as it enables trainee students to cope with difficulties based on theoretical ground and become capable of surmounting linguistic, cultural and ideological hurdles. The last group of participants who use translation theory in their training base their use on the notion that theory and practice should go hand in hand. They argue that theory helps trainee students know what they are doing, the reason behind their action and it is often embedded in practice unless highlighted by instructors/trainers. It is but the theory that frames trainee students’ experiences and practices. They conclude that translation theory is an essential part of translation education that is inseparably linked to practice.

**Table 5:** The percentage of the participants’ use of translation theory in the translation/interpreting training.

Participants’ use of translation theory	Percentage
The participants who use translation theory in the translation/interpreting training	86.7%
The participants who do not use translation theory in the translation/interpreting training	13.3%

Conversely, the minority of the participants do not make use of translation theory in the translation/interpreting training in EMI higher education classes in the selected Arab countries. One group of them are of the view that translation is considered a practical activity and has no theoretical basis. Another group believe that not all instructors/trainers have academic background and research interests to learn and use theories in their training.

Table 6 shows that 46.2% of the participants always use translation theory in the translation/interpreting training in EMI higher education classes in the selected Arab countries. One group of them state that instructors/trainers usually tend to comprehend processes when mingled with a theoretical framework prior to the practical training. Another group point out that it is crucial for trainee students to understand that translation/interpreting is deemed an applied science. They continue to argue that trainee students should be accustomed to applying the theories they have received in their training. They further add that the main goal for using theories in training is that the target text should be produced idiomatically as if it were originally written in the target language. A third group of participants believe that translation/interpreting students are required to reflect the use of idioms, expressions and cultural terms that convey the intended meaning. They go on to explicate that evidence for theory should be given from practice and vice versa. They conclude that training on translating/interpreting any particular text needs always both theory and practice.

**Table 6:** The percentage of the frequency of the participants’ use of translation theory in the translation/interpreting training.

Frequency of the participants’ use of translation theory	Percentage
Always	46.2%
Often	23.1%
Sometimes	26.9%
Little	3.8%
Seldom	0%

On the other hand, about 23.1% of the participants often use translation theory in the translation/interpreting training in EMI higher education classes in the selected Arab countries. One group of them claim that translation theory helps sharpen trainee students’ skills. Another group of participants believe that theories enable trainee students to weigh and take decisions based on the translational situation and context. Furthermore, they assert that practice needs theoretical foundation to foster translation/interpreting training. About 26.9% of the participants sometimes use translation theory in the translation/interpreting training in EMI higher education classes in the selected Arab countries. One group of them argue that the use of translation theory in training depends chiefly on the level of the trainee students. They further add that theories are resorted to when translating/interpreting terms. Another group of participants hold the view that the use of translation theory in student training relies mainly on the translation/interpreting students’ educational level. They explain that in training, there is a focus on methods and skills required for practical translation which also comprises theory. They conclude that the use of translation theory in student training is principally built upon the training situation and context. About 3.8% of the participants slightly use translation theory in the translation/interpreting training in EMI higher education classes in the selected Arab countries. No record of any participant who seldom uses translation theory in the translation/interpreting training in EMI higher education classes in the same countries.

As shown in Table 7, the participants whose use of translation theory is contingent upon text type are 83.3%, whereas those whose use of translation theory is not contingent upon text type are 16.7%. Hence the majority of the participants use translation theory on the basis of text type in training students to be translators/interpreters in EMI higher education classes in the selected Arab countries. One group of them point out that the use of translation theory is dependent on the text type itself. They claim that each text type requires a particular terminology, audience, design and formula. They go on to contend that different translation theories work properly with different text types. Hence the use of a particular translation theory is evidently influenced by the type of text which is subject to translation. Another group of participants hold the view that not all translation theories can be applied to all types of text. They continue to assert that the use of translation theory hinges upon text type and methods used for translation. A third group of participants, adding value to what has been said by scholars on the type of text that needs to be translated, emphasize the importance of reviewing the literature specific to the type of text that requires translation prior to applying a particular theory thereto. They argue over the merit of the *de facto* impact of text type on the selection of translation theory. The last group of participants who believe that the use of translation theory is contingent upon text type state that instructors/trainers should have a clear vision concerning the translation theory(s) that should be applied to the text that demands translation. They claim that text and context have a major role to play with regard to the specification of the translation theory(s) that is needed for application. On the contrary, the minority of the participants are of the opinion that the use of theory is not contingent upon text type in training students to be translators/interpreters in EMI higher education classes in the selected Arab countries. They argue that no matter what type of text is given for translation, the instructor/trainer is required to have a certain theory to be applied to his/her translation/interpreting, thus resulting in producing the closest natural equivalent translation.

**Table 7:** The classification of the participants’ uses of translation theory on the basis of text type and their percentages.

Classifying participants’ use of translation theory on the basis of text type	Percentage
The participants whose use of translation theory is contingent upon text type	83.3%
The participants whose use of translation theory is not contingent upon text type	16.7%

As Table 8 shows, 59.3% of the participants use the interpretive theory of translation in their translation/interpreting training in EMI higher education classes in the selected Arab countries, while 40.7% of them utilize text-linguistics in their training. About 37% of the participants adopt contrastive analysis in their translation/interpreting training in EMI higher education classes in the same countries, whereas 33.3% of them make use of information processing models in their training. Around 29.6% of the participants employ literal translation in their translation/interpreting training in EMI higher education classes in the selected Arab countries, whereas 25.9% of them do use other theories beside the ones mentioned above in their training. It is worth noting that the majority of the participants adopt multiple theories, i.e., they employ more than a single theory in their translation/interpreting training in EMI higher education classes in the selected Arab countries. On the contrary, the minority of the participants rely on a single theory in their translation/interpreting training. For instance, 16.67% of 59.3% of the participants who use the interpretive theory of translation in their translation/interpreting training, adopt it on its own, while 10% of 33.3% of the participants who employ information processing models in their training, use it exclusively. About 3.34% of 40.7% of the participants who utilize text-linguistics in their translation/interpreting training, use it individually. The same percentage of 25.9% of the participants who have chosen ‘other’, specify their used theory in the translation/interpreting training as skopos theory. They claim that both the scope of the required task and the intended audience play a major role in determining the theory(s) that should be used in the translation/interpreting training.

**Table 8:** The translation theories used by the participants in the translation/interpreting training and their percentages.

Translation theories used	Percentage
Literal translation	29.6%
Contrastive analysis	37%
Text-linguistics	40.7%
Information processing models	33.3%
The interpretive theory of translation	59.3%
Other	25.9%

By contrast, the majority of the participants make use of multiple theories in their translation/interpreting training in EMI higher education classes in the selected Arab countries, as stated earlier. One group of them adopt all the theories given in Table 8: literal translation, contrastive analysis, text-linguistics, information processing models and the interpretive theory of translation. They have also chosen ‘other’, specifying their other used theory as communicative translation. They justify their chosen options by stating that it is crucial to expose trainee students to a variety of different theories and train them on how to use such theories. Another group of participants also use all the theories mentioned above: literal translation, contrastive analysis, text-linguistics, information processing models and the interpretive theory of translation. A third group of participants use in their training contrastive analysis, text-linguistics, information processing models and the interpretive theory of translation. Moreover, they have picked ‘other’, specifying their other used theories as functional theories, cultural turn as well as polysystem theories. Another group of participants adopt literal translation, text-linguistics and the interpretive theory of translation. They have also chosen ‘other’, specifying their other used theories as skopos theory and decision-making techniques. They point out that each translation theory has its own use. For instance, in legal translation, instructors/trainers tend to adopt literal translation, while other types of translation may demand more adaptation. They further add that instructors/trainers should inform trainee students of the difference between translation/interpreting practice and other related administrative work, such as translation editing.

A fifth group of participants utilize contrastive analysis, text-linguistics, information processing models and the interpretive theory of translation. Two groups of participants employ contrastive analysis, text-linguistics and the interpretive theory of translation in their training. They believe that trainee students should receive 30% of theories and 70% of practice in their translation/interpreting training. They explain the reason behind their use of contrastive analysis, stating that it is of utmost significance to show trainee students the similarities and differences between the languages used in their translation/interpreting training. They then justify their use of text-linguistics by asserting that it is important that instructors/trainers show trainee students how to keep their translation/interpreting within the original context, preserving the form and content of the text concerned. With regard to their use of the interpretive theory of translation, they state that translation/interpreting students are required to understand the source language text and fully grasp the intention relayed by the source language text. An eighth group of participants use text-linguistics and information processing models. They have also selected ‘other’, specifying their other used theory as communicative approach. They hold the view that instructors/trainers should train their trainee students to use different theories together as no single theory has proved to be the only effective theory. Another group of participants make use of text-linguistics and the interpretive theory of translation. Furthermore, they have picked ‘other’, specifying their other used theory as sociolinguistic approach. They argue over the merit of the necessity of striking a balance between preserving both the form and content of the source text in the target text and producing an idiomising translation, taking into account the social context of the target language culture.

Two other groups of participants adopt literal translation and contrastive analysis in their training. They claim that these two theories are important in translation/interpreting training. Trainee students should know how to apply them to their translation/interpreting practice. Two different groups of participants adopt literal translation and the interpretive theory of translation. They defend their choices by asserting that literal translation is indispensable in translating titles, certain terms, etc. They go on to claim that literal translation should be used in training with its three types for different purposes. These types are: word-for-word, one-to-one and translating the meaning literally, with more emphasis placed on the latter. They further add that other theories can also be adopted in training, such as free translation, pragmatic translation as well as communicative translation. Another group of participants adopt literal translation and information processing models. They support their choice by claiming that both the aforementioned theories scaffold the translation/interpreting students in transferring the appropriate meaning. Hence they need to be used in the translation/interpreting training. A different group of participants employ contrastive analysis and text-linguistics in their training. Another group of participants adopt contrastive analysis and the interpretive theory of translation in the training. The last group of participants who believe in the use of more than a single theory in the translation/interpreting training pick ‘other’, explaining their choice by pointing out that they make use of multiple theories based on the text type, type of audience as well as cultural considerations.

As shown in Table 9, 100% of the interviewees use translation theory in the translation/interpreting training. In other words, all the three full professors instructors/trainers interviewees who have participated in both questionnaire and interviews make use of translation theory in training postgraduate students to be translators/interpreters in EMI higher education classes in their countries. One of them states: “at a postgraduate level, there is no way students can translate or interpret without theoretical foundation to back their translation/interpreting decision-making.” Another interviewee, supporting the views of the previous one, claims that postgraduate students should be able to justify their translation/interpreting choices through identifying the approaches, methods, procedures, techniques and strategies they adopt upon embarking on any translation/interpreting project. This will never be accomplished if students are not familiar with at least the most prominent translation theories and are abreast of how to apply them to their practical translation/interpreting work. Theories serve as a guiding tool for the sake of improving and justifying practice. She also admits that there may be theories the application of which is not possible, which makes them practically pointless. She concludes that postgraduate students who are trained in translation/interpreting should be conversant with translation theories and should apply them to their translation/interpreting practice to improve their work and distinguish themselves from other translators/interpreters who have no knowledge of translation/interpreting studies.

**Table 9:** The percentage of the interviewees’ use of translation theory in the translation/interpreting training.

Interviewees’ use of translation theory	Percentage
The interviewees who use translation theory in the translation/interpreting training	100%
The interviewees who do not use translation theory in the translation/interpreting training	0%

The last interviewee, lending credence to the claims made by the previous two interviewees, points out that if postgraduate students do not use translation theory in their translation/interpreting practice, there will be no benefit from studying translation theory, and placing it as a core course in the majority of MA translation/interpreting programs that are offered in the selected Arab countries will be useless. He contends that instructors/trainers in the countries under study should explain the significance of utilizing translation theory in translation/interpreting practice. Also, the interviewee explains that he makes use of translation theory in training his postgraduate students to translate/interpret to enhance their knowledge of translation/interpreting studies and concurrently creates a solid link between theory and practice. He goes on to claim that translation theory as a core course in translation/interpreting postgraduate programs in the countries under study should be viewed and dealt with by Arab instructors/trainers in two distinct ways; it should be taught as pure theoretical knowledge when studied as an autonomous course, while it should be exploited and applied to translation/interpreting practice during practical training. The latter, indeed, shows the instructor/trainer whether or not his/her students have intrinsically comprehended the former. The interviewee concludes that instructors/trainers should show the students the difference between translating/interpreting with the use of translation theory and translating/interpreting without its use to further encourage them to apply the appropriate theory to their translation/interpreting practice.

Table 10 shows that 100% of the interviewees practice translanguaging when using translation theory in the translation/interpreting training. In other words, all the three full professors instructors/trainers interviewees who have participated in both questionnaire and interviews exercise translanguaging when using translation theory in training postgraduate students to be translators/interpreters in EMI higher education classes in their countries. One of the interviewees states: “I find using the first language in EMI postgraduate classes, particularly in showing students how to use translation theory when they translate/interpret a particular text both necessary and useful. It is necessary in the sense that students may come across specific theoretical translation concepts that are difficult to understand using English only. Translanguaging is also useful in EMI higher education classes in training students to utilize translation theory when practicing translation/interpreting as it helps store and retain information easily.” The second interviewee holds the view that Arabic needs to be used in EMI postgraduate classes in certain situations; one of which is when instructors/trainers show their students during the translation/interpreting training how to apply translation theory. He goes on to claim that there are multiple theories within the realm of translation and interpreting studies, the cognisance of which cannot be expected from all translation/interpreting students. What is more, such translation theories may contain specific terminology which cannot be easily handled by students with the use of a foreign language on its own. Hence translanguaging may serve as an effective solution to surmount such hurdles. He concludes that new research supports the use of first language in foreign language-instructed classes provided that the former is only resorted to in certain linguistic situations and limited instances.

**Table 10:** The percentage of both the interviewees who completely adhere to Englishisation and those who practice translanguaging when using translation theory in the translation/interpreting training.

Use of Englishisation only versus translanguaging	Percentage
The interviewees who completely adhere to Englishisation when using translation theory in the translation/interpreting training	0%
The interviewees who practice translanguaging when using translation theory in the translation/interpreting training	100%

The last interviewee is of the belief that getting through to the students should be taken as the first priority regardless of the linguistic medium through which theoretical knowledge is transmitted to students. In EMI postgraduate translation/interpreting classes, instructors/trainers often make sure that information is conveyed in English. However, when using translation theory within the translation/interpreting training context, translanguaging may be resorted to for the sake of simplifying the explanation of a particular theory and facilitating the application thereof. She admits that her students understand theories and apply them in a better way when explained in both English and some Arabic rather than when explained purely in English. She continues to assert that one of her best students, albeit fluent in English, informs her that she prefers translanguaging over Englishisation when receiving detail of translation theory. The student points out, she adds, that she feels more comfortable with translanguaging and performs confidently using translation theory in translation/interpreting practice. The interviewee concludes that with training in general, and with the translation/interpreting training in particular, instructors/trainers should utilize the optimum training method by which trainee students can receive the appropriate training in a very comfortable way, including the language(s) used in the training. The more comfortable the trainee students are, the more effective the training is.

## 7. Discussion

As an answer to the first research question of the present paper, all the interviewees and the majority of the participants who have taken part in the questionnaire use translation theory in the translation/interpreting training in EMI postgraduate classes in the selected Arab countries. They hold the view that translation theory serves as a solid basis on which trainee students can build their decision-making and problem-solving. This runs in line with Lederer (2007), who claims that translation theory plays a substantial role in systematizing and generalizing translation problems. The participants consider translation theory a fruitful guide for trainee students, which provides them with the right direction to undertake the translation process successfully. This notion is supported by Ingo (1992), who compares the importance of the role played by translation theory with regard to translation/interpreting to that of the role fulfilled by grammar concerning languages. Lederer (2007), supporting this concept, points out that translation theory can be employed by instructors/trainers to guide trainee students to think properly and provide them with specific rules that can assist them to make strategic decisions. Also, the interviewees claim that postgraduate students should not translate or interpret without theoretical basis on which they base their translation/interpreting practice. Students should be conversant with the major translation theories and how they are applied to translation practice. This goes hand in hand with Wilss (1998), who asserts that the theoretical framework in the translation/interpreting training helps strengthen trainee students’ declarative as well as procedural knowledge.

As a response to the second research question of the current research, all the interviewees practice translanguaging when using translation theory in the translation/interpreting training in EMI postgraduate classes in their countries. Some interviewees are of the opinion that the use of translanguaging is necessary when translation theories contain certain terms with concepts that are difficult to understand by trainee students with the use of English as the only medium of instruction. This is advocated by Cook (2001), who supports the use of the mother tongue in teaching second/foreign languages, though the latter should be used extensively. Other interviewees hold the view that translanguaging is useful in storing, maintaining and recalling information specific to translation theory within the translation/interpreting training context. This is given credence by García (2008), who argues over the merit of exercising translanguaging in classrooms. Lucas and Katz (1994) and Cummins (2001, 2007, 2009), backing this theme, claim that since English learners’ preexisting knowledge is encoded in their mother tongue, building on this knowledge requires relating new concepts in English to the learners’ first tongue cognitive schemata. Such linking demands the use of the learners’ first language in the classroom to be properly accomplished. Other interviewees assert that their postgraduate students prefer translanguaging over Englishisation when listening to the detail of translation theory within the context of translation/interpreting training. They report that one of their best students, albeit fluent in English, points out that she feels more comfortable when receiving detail about translation theory through translanguaging for the purpose of translation/interpreting practice. This notion is in line with Malakoff and Hakuta (1991), who contend that translation helps raise linguistic awareness and promote bilingualism in such a flexible method.

Answering the third research question of the paper concerned, the majority of the participants always use translation theory in the translation/interpreting training in postgraduate classes in the selected Arab countries. This points to the importance and usefulness of the use of translation theory in training postgraduate students to be translators/interpreters in the countries under study. It fosters the link between translation theory and practice and, at the same time, lends credence to the notion that theories have been formulated to be implemented. Such finding promotes the practical application of translation theories and encourages Arab instructors/trainers and other instructors/trainers to place more emphasis on the use of translation theories in their postgraduate as well as undergraduate training and encourage their MA as well as BA students to use them in their translation/interpreting practice.

Responding to the fourth research question of the research in question, the majority of the participants adopt multiple theories in their translation/interpreting training in postgraduate classes in the selected Arab countries. Among the theories that they adopt are: literal translation, contrastive analysis, text-linguistics, information processing models, the interpretive theory of translation, communicative translation, skopos theory, polysystem theory, functional theory, cultural turn and sociolinguistic theory. The most commonly used theory is the interpretive theory of translation, which is exploited by about 59.3% of the participants. Such use of multiple translation theories in the translation/interpreting training process may be justifiable, taking into consideration the *de facto* differences in text types, translation purposes and types of target audience. Even within a single particular text, the translator may need to use a diverse set of theories to arrive at an acceptable, yet adequate translation. This conclusion has been reached by Alwazna (2014b) within the context of translating the Ottoman Majalla and the translation strategies used for this purpose where he points out that in legal translation, a single small text may warrant a variety of different translation strategies based on different factors, such as the difference in legal rules governing the source legal system and the target legal system, the difference in the legal language specific to each legal system and the type of readership. Based on the foregoing, the translator and the translation/interpreting instructor/trainer would unequivocally require multiple translation theories to undertake the training process successfully, addressing each text on its own merit. This would undoubtedly promote students’ understanding of different text types and raise their linguistic awareness in such a way that enables them to understand each translation theory, know how it is used and adopt it in the appropriate linguistic situation.

## 8. Conclusion

The use of translation theory had been and is still a debatable topic within the context of translation/interpreting training. Two opposing views of translation scholars and professional translators regarding the use of translation theory in the translation and interpreting training are found. At one end of the spectrum, there are the views of those who do not see the use of translation theory necessary in the translation and interpreting training. They argue that the translation/interpreting students need to join the profession and spend some time practising. At the other end of the spectrum, there are those who believe in the use of translation theory in the translation and interpreting training as translation theory is fruitful in regulating and generalizing translation problems, directing trainee students to proper and deep thinking and offering certain principles that may scaffold trainee students in the process of decision-making.

The present paper argues that all the interviewees and the majority of the participants who have taken part in the questionnaire make use of translation theory in the translation/interpreting training in EMI higher education classes in the selected Arab countries. These Arab instructors/trainers use theory in their training as it helps trainee students accomplish their practical translation tasks properly. It serves as a guide for trainee students to undertake processes of problem-solving and decision-making. The paper also shows that all the interviewees do not completely adhere to English as the medium of instruction in using translation theory when training students to be translators/interpreters in EMI higher education classes in their countries, rather they exercise translanguaging. This is owing to the fact that certain translation theories are complicated and cannot be comprehended by trainee students through Englishisation only. The paper further demonstrates that the majority of the participants use translation theory in the translation and interpreting training, i.e., about 46.2% of them always use translation theory in their training in EMI higher education classes in the selected Arab countries. Finally, the present paper claims that the majority of Arab trainers use multiple theories in the translation/interpreting training in EMI higher education classes in the same countries. The participants justify their multiple use of theories by stating that each theory has its own use and is adopted for a specific purpose. This paper is limited to addressing the use of translation theory by Arab instructors/trainers in the translation/interpreting training in EMI higher education classes within the selected Arab countries in the Middle East and whether Englishisation or translanguaging is adopted in using translation theory in such training. Further research is required to examine the use of translation theory by other instructors/trainers, such as Europeans, Americans, Africans, and so on in the translation/interpreting training in EMI higher education classes in their countries and whether Englishisation or translanguaging is made use of to see whether or not the research will yield the same results. A comparative study can then be conducted to see the similarities and differences between the results of the current research and those of the recommended one. Further endeavors are needed to investigate the reasons behind the use of the interpretive theory of translation by the majority of the participants as this theory scores the highest number of uses by Arab instructors/trainers. Research can also be carried out to question whether or not Arabic has any impact on the use of any of the theory(s) employed and whether or not some theories work better with Arabic than with other languages. Similar research is required to question the views of professional translation/interpreting practitioners as to whether or not they use translation theory in their training and whether or not they use English only or translanguaging in their training.

## Data availability statement

The original contributions presented in the study are included in the article/supplementary material, further inquiries can be directed to the corresponding author.

## Ethics statement

Ethical review and approval was not required for the study on human participants in accordance with the local legislation and institutional requirements. Written informed consent from the [patients/participants OR patients/participants legal guardian/next of kin] was not required to participate in this study in accordance with the national legislation and the institutional requirements.

## Author contributions

The author confirms being the sole contributor of this work and has approved it for publication.

## Funding

This project was funded by the Deanship of Scientific Research (DSR) at King Abdulaziz University, Jeddah, under grant No. (G: 27–125-1443). The author, therefore, acknowledges with thanks DSR for technical and financial support.

## Conflict of interest

The author declares that the research was conducted in the absence of any commercial or financial relationships that could be construed as a potential conflict of interest.

## Publisher’s note

All claims expressed in this article are solely those of the authors and do not necessarily represent those of their affiliated organizations, or those of the publisher, the editors and the reviewers. Any product that may be evaluated in this article, or claim that may be made by its manufacturer, is not guaranteed or endorsed by the publisher.
